# Exosomes: Emerging Insights into the Progression of Pancreatic Cancer

**DOI:** 10.7150/ijbs.97076

**Published:** 2024-08-01

**Authors:** Xulin Zhou, Yongmin Yan, Ye Shen, Min Xu, Wenrong Xu

**Affiliations:** 1 Department of Gastroenterology, Affiliated Hospital of Jiangsu University, Zhenjiang 212001, China.; 2 Department of Laboratory Medicine, Wujin Hospital Affiliated with Jiangsu University, Changzhou, 213000, China.; 3 Department of Hepatobiliary Pancreas Surgery, Aoyang Hospital Affiliated to Jiangsu University, Suzhou, 215000, China.; 4 Jiangsu University School of Medicine, Jiangsu University, Zhenjiang 212001, China.

**Keywords:** exosomes, pancreatic cancer, biomarker, progression

## Abstract

Pancreatic cancer is a very aggressive and fatal malignancy with few therapeutic choices and a poor prognosis. Understanding the molecular pathways that drive its growth is critical for developing effective therapeutic strategies. Exosomes, small extracellular vesicles secreted by numerous cell types, have recently emerged as essential intercellular communication mediators, with implications for tumor growth and metastasis. In this article, we present a review of current knowledge about exosomes and their role in pancreatic cancer progression We discuss the biogenesis and characteristics of exosomes, as well as their cargo and functional significance in tumor growth, immune evasion, angiogenesis, invasion, and metastasis. We further emphasize the potential of exosomes as diagnostic biomarkers and therapeutic targets for pancreatic cancer. Finally, we discuss the challenges and future perspectives in using exosomes to improve patient outcomes in pancreatic cancer.

## Introduction

Cancer is a severe threat to human health, killing approximately 600,000 individuals per year 1. Among them, pancreatic ductal adenocarcinoma stands out as a highly dangerous tumor of the digestive tract, ranking as the fourth leading cause of cancer-related deaths worldwide. Unfortunately, the five-year survival rate for patients diagnosed with this type of cancer is less than 9% 2. Currently, surgical treatment remains the primary approach for treating pancreatic cancer. However, the lack of early specific methods has resulted in missed opportunities for optimal timing. 3. Both environmental and genetic factors play crucial roles in the development of pancreatic cancer (PC) 4. In fact, during the progression of PC, various factors can contribute to its malignant behavior. Understanding the interactions between these factors is a prominent focus in the study of PC's malignant behavior and related mechanisms.

Extracellular vesicles, emitted by a variety of cellular sources, are typically categorized into three distinct types: apoptotic bodies, microvesicles, and exosomes. This classification is based on the differences in their size, molecular content, and the mechanisms by which they are formed 5. Exosomes, in particular, stand out among other extracellular vesicles for their role in mediating cell-to-cell communication, playing a pivotal part in the exchange of signals and substances between cells 6. The inward folding of the plasma membrane gives rise to a cup-shaped structure, which aids in the creation of an early-sorting endosome (ESE). This ESE undergoes maturation and develops into late-sorting endosomes (LSEs), which produce multivesicular bodies (MVBs). Intraluminal vesicles (ILVs) are enclosed within these MVBs' lumens before being released as exosomes 7, 8. These exosomes have a diameter ranging from 30 to 150nm and contain proteins, nucleic acids, and lipids 9. The unique lipid bilayer structure of exosomes originating from the inward folding of the plasma membrane allows them to protect their contents from external factors while still easily crossing cell membranes. This enables the substances carried by exosomes to be more efficiently absorbed by recipient cells 10.

At present, the application of exosomes as potential early molecular markers and drug delivery vehicles has garnered significant interest among researchers. The ability to isolate and purify exosomes is essential for successful research and development. Luckily, with advanced methodologies such as sequential ultracentrifugation, gradient ultracentrifugation, and ultrafiltration, this process has been greatly facilitated 11. Furthermore, electroporation enables exosomes to encapsulate small molecule substances and therapeutic agents, thereby broadening the clinical applications of engineered extracellular vesicles 11. In the context of pancreatic cancer (PC), both cancer-derived and non-cancer-derived exosomes play a significant role in promoting the progression of the disease. They can worsen the prognosis of patients by influencing PC cell behavior, modifying the tumor microenvironment, and increasing invasiveness and resistance. Notably, exosomes serve as a biomarker in liquid biopsy for patients with pancreatic cancer, enabling early prediction before the manifestation of significant symptoms. Additionally, the engineering exosomes facilitate more efficient drug delivery to pancreatic cancer tissues. Therefore, understanding the role of exosomes in PC research can provide valuable insights and pave the way for future investigations in this field.

## Cargo Composition of Exosomes in Pancreatic Cancer

**Figure 1:**
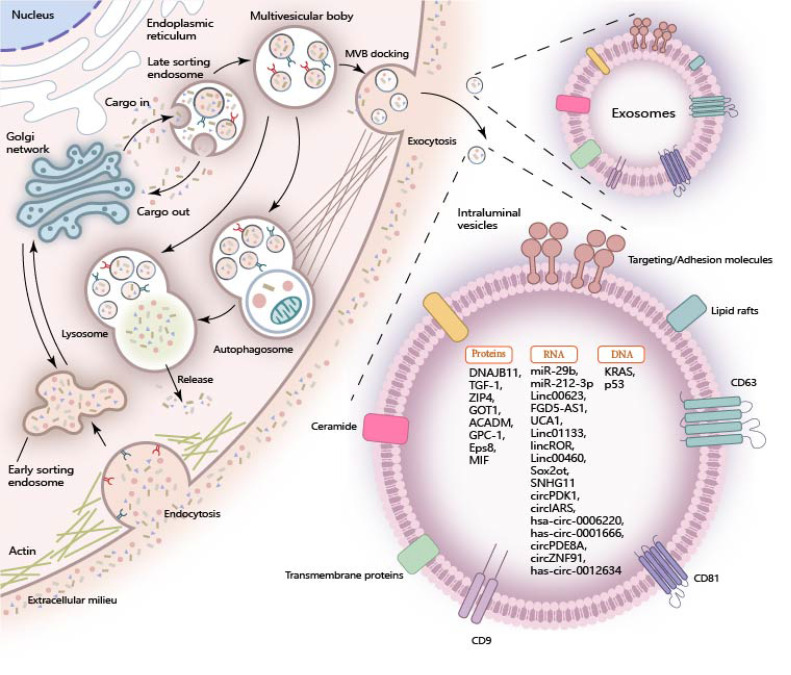
** Cargo of Exosomes in Pancreatic Cancer.** During the biogenesis of exosomes, the inward folding of the plasma membrane and the sequential development of early-sorting endosomes (ESE), late-sorting endosomes (LSEs), and multivesicular bodies (MVBs) are pivotal steps. These processes enable the sequestration of a wide array of molecular constituents, including proteins, RNA, and DNA, into the exosomal lumen. Once internalized by target recipient cells, these encapsulated cargo molecules exert significant influence over a variety of cellular processes, thereby participating in intercellular communication and function regulation.

Exosomes are formed through the invagination of the plasma membrane and transport cell-surface proteins, soluble proteins, and small molecules like lipids, nucleic acids, and proteins. The encapsulated cargoes play a crucial role in regulating diverse metabolic processes within pancreatic cancer cells when they are internalized by recipient cells through exosomal uptake (Figure 1).

### Exosomal RNAs in Pancreatic Cancer

Exosomal RNA plays a pivotal role in intercellular communications, particularly in the context of pancreatic cancer. Specifically, exosomal miRNAs, lncRNAs, and circRNAs play a crucial role in regulating the progression of pancreatic cancer. MiRNA is a class of noncoding transcripts comprising 18-23 nucleotides, which govern gene protein expression by binding to the 3′UTRs of mRNA molecules 12. These exosomal miRNAs from PC cells are actively transferred to recipient cells, thus exerting a profound influence on the development of pancreatic cancer. For instance, exosomal miR-29b is delivered into human umbilical vein endothelial cells (HUVECs) and attenuates angiogenesis in pancreatic cancer 13. Similarly, exosomal miR-212-3p is delivered to dendritic cells (DCs) and inhibits the expression of RFXAP, thereby disrupting the immune tolerance of dendritic cells 14. LncRNA is a diverse class of transcripts longer than 200 nucleotides that possess minimal or no protein-coding ability 15. Similar to miRNAs, exosomal lncRNAs function as crucial regulators in the progression of pancreatic cancer. Notable examples include exosomal LINC00623 16, FGD5-AS1 17, UCA1 18, LINC01133 19, linc-ROR 20, LINC00460 21, Sox2ot 22, SNHG11 23. CircRNAs represent a class of non-coding RNAs characterized by their covalently closed loop structure, which confers enhanced stability and resistance to degradation 24. Within exosomes derived from pancreatic cancer (PC) cells, circRNAs are found in high abundance and are known to perform a myriad of functions. Notable examples include circPDK1 25, circ-IARS 26, hsa_circ_0006220, has_circ_0001666 27, circ-PDE8A 28, circZNF91 29, and has_circ_0012634 30, each contributing uniquely to the complex regulatory network in PC. These exosomal circRNAs have been implicated in regulating the proliferation and migration of pancreatic cancer.

### Exosomal Proteins in Pancreatic Cancer

Exosomal proteins in pancreatic cancer can be classified into two main categories. The first category comprises exosomal structural proteins, which include components of the cytoskeleton, as well as proteins involved in membrane fusion and transport. These proteins are strategically located on the exosomal surface and within the exosomal lumen, playing crucial roles in the exosome's structure and function. Characteristic proteins such as Annexins, Flotillin, Alix, and TSG101 31 are frequently utilized for the identification and characterization of exosomes, given their presence is indicative of exosomal composition and function. The other proteins are directly involved in the regulation of proliferation, migration, and invasion through multiple signal pathways in pancreatic cancer. Notably, certain exosomal proteins have been identified for their specific roles in pancreatic cancer progression. For instance, exosomal DNAJB11 has been reported to regulate the development of pancreatic cancer via the EGFR/MAPK pathway 32. Exosomal TGF-β1 is implicated in the induction of epithelial-mesenchymal transition (EMT) in pancreatic cancer 33. Furthermore, exosomal ZIP4 enhances the proliferation of pancreatic cancer 34. The upregulated expression of GOT1 in pancreatic cancer tissues is remarkable, as exosomal GOT1 has been found to suppress ferroptosis in pancreatic cancer by activating the Nrf2/HO-1 axis 35. Moreover, exosomal proteins are associated with chemotherapy resistance and hold potential as biomarkers for pancreatic cancer. For instance, exosomal ACADM has been correlated with gemcitabine sensitivity and may serve as a predictive biomarker for chemotherapy response in patients 35. Glypican-1 (GPC1) is found to be overexpressed in the exosomes of pancreatic cancer patients compared to those of healthy individuals and individuals with benign pancreatic diseases, suggesting that exosomal GPC1 could potentially serve as a biomarker to aid in the diagnosis and stratification of pancreatic cancer 36. Eps8 has been observed to be highly expressed in exosomes from pancreatic cancer cells with metastatic characteristics, metastasis‑derived, indicating that exosomal Eps8 holds promise as a biomarker for pancreatic cancer metastasis 37. Macrophage migration inhibitory factor (MIF) is also reported to be highly expressed in the PC-derived exosomes. Exosomal MIF favors the liver for metastasis and may serve as a prognostic marker for the development of PDAC liver metastasis 38.

### Exosomal DNAs in Pancreatic Cancer

Similar to other substances found in exosomes, exosomal DNAs have shown greater potential in diagnostic applications. These DNAs can be detected more easily in the circulatory system when carried by exosomes, serving as biomarkers for diagnosis and prognosis. Among the most common mutant genes in pancreatic cancer, KRAS mutations are frequently observed. In the serum exosomes of pancreatic cancer patients, KRAS mutations in exosomes have been detected in 43.6% of early-stage cases 39. TP53 mutation, another common mutation in pancreatic cancer has also been identified in exosomes derived from pancreatic cancer, highlighting its potential as a biomarker for this disease 40, 41. Furthermore, the application of engineered exosomes has facilitated the impact of exosomal DNA on the progression of pancreatic cancer. Researchers have documented the innovative application of non-autologous exosomes for the delivery of CRISPR/Cas9 plasmid DNA, specifically aimed at targeting the mutant Kras G12D oncogenic allele within pancreatic cancer cells. This approach has demonstrated the potential to effectively suppress tumor growth, offering a promising therapeutic strategy in the treatment of pancreatic cancer 42. However, further exploration is needed to fully understand and optimize this approach.

## Functional Implications of Exosomes in the Progression of Pancreatic Cancer

**Figure 2:**
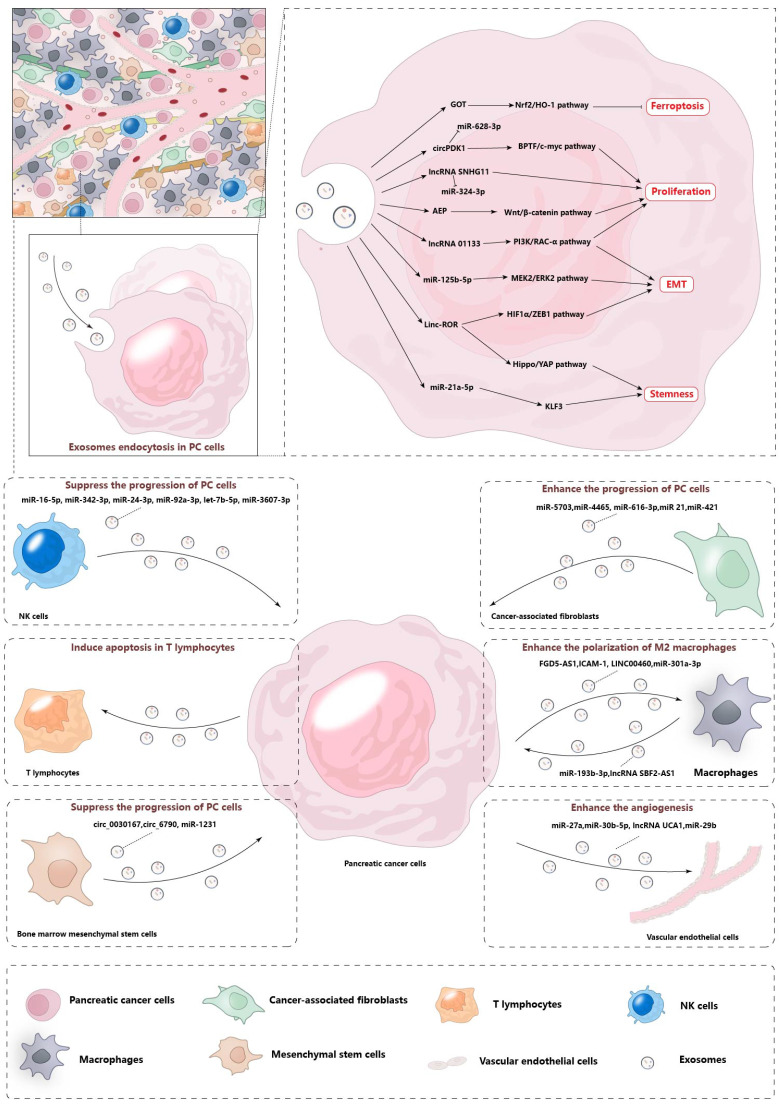
** Pancreatic cancer cells regulate microenvironmental remodeling by secreting exosomes.** There are a variety of stromal cells in the microenvironment of pancreatic cancer, such as CAF, macrophages, endothelial cells, etc. Pancreatic cancer cells interact with these cells by secreting exosomes and regulating related biological functions. In addition, other stromal cells also affect tumor cells through the secretion of exosomes, which promotes the remodeling of the pancreatic cancer microenvironment.

The advancement of pancreatic cancer (PC) encompasses a complex interplay of processes, including the proliferation, invasion, stem-like characteristics, and drug resistance of malignant cells. These phenomena are orchestrated by a multitude of regulatory factors that influence the trajectory and severity of the disease. Exosomes from various sources directly or indirectly favor these malignant tumor behaviors. They can be roughly divided into two types. One type is derived from cancer cells, and these exosomes are consumed by their cancer cells or neighboring cancer cells after being released, thus causing effects. The other types of exosomes come from non-cancer cells, including stromal cells, immune cells, and endothelial cells. These exosomes not only impact the cancer cells themselves but also influence the biological behavior of surrounding cells, thereby modifying the tumor microenvironment to enhance tumor growth. Exosomes act as critical mediators of intercellular communication, linking tumor cells with their surrounding microenvironmental cells. This interaction, as illustrated in Figure 2, plays a pivotal role in the promotion of tumorigenesis by modulating the local environment to support cancer cell growth and progression.

### Modulation of the tumor microenvironment

The tumor microenvironment plays an important role in tumor progression. In the case of pancreatic cancer, different types of cells within the tumor immune microenvironment interact with each other, such as immune cells, endothelial cells, and fibroblasts. These interactions lead to the development of unique immunosuppressive properties within the pancreatic cancer microenvironment. Additionally, the tumor microenvironment also has a regulatory effect on the malignant behavior of PC cells. Exosomes, which act as carriers for intercellular message transmission, play an important role in regulating the PC microenvironment. Exosomes derived from pancreatic cancer are responsible for activating various cells within the microenvironment, and these activated stromal cells support the progression of tumor cells.

#### Exosomes and PC macrophages

Macrophages are an important type of stromal cells found in the PC microenvironment. These cells are derived from progenitor cells in bone marrow. Macrophages can be divided into two subtypes: M1 and M2. Polarized M1 macrophages, which are induced by IFN-γ or lipopolysaccharides, play a role in the immune response of type I helper T cells to different pathogens. They produce pro-inflammatory cytokines and can phagocytose tumor cells. On the other hand, M2 macrophages undergo polarization in response to cytokines such as IL-4, IL-10, or IL-13. They express a distinct set of molecules including arginase 1, IL-6, and angiogenic factors, all of which contribute significantly to the processes of tumorigenesis by creating a supportive microenvironment for tumor growth and progression 43. Exosomes derived from PC cells deliver FGD5-AS1 into macrophages, leading to the polarization of M2 macrophages and promoting the growth of pancreatic cancer. FGD5-AS1 interacts with p300, resulting in the acetylation and activation of STAT3, as well as the activation of the STAT3/NF-κB pathway 17. Additionally, ICAM-1, which is enriched in pancreatic cancer cells and their exosomes, interacts with CD11c on macrophages to regulate them. Exosomal ICAM-1 increases the levels of surface markers that indicate polarization towards an immunosuppressive M2-like phenotype.^44^. The expression of LINC00460 is increased in both PC cells and tissues. Exosomal LINC00460 promotes the polarization of M2 macrophages and supports the growth of tumor cells 21. In a hypoxic state, miR-301a-3p is highly expressed in PC cells. Hypoxic exosomal miR-301a-3p from PC cells induces the polarization of M2 macrophages through the activation of the PTEN/PI3Kγ signaling pathway 45. In conclusion, exosomes derived from PC cells play a role in the polarization of M2 macrophage. Interestingly, M2 macrophages secret exosomes containing miR-193b-3p 46 and lncRNA SBF2-AS1 47. These exosomes have been reported to suppress the malignant growth of PC cells, despite M2 macrophages typically being considered as immune cells that promote cancer. Further research is needed to explore whether exosomes derived from M2 macrophages can be a breakthrough in the treatment of PC.

#### Exosomes and Vascular Endothelial Cells in PC

The enriched vascular system is a key factor in the metastasis of pancreatic cancer 48. Human vascular endothelial cells (HUVECs) play a crucial role in promoting the growth of new blood vessels of PC. Pancreatic cancer-derived exosomes are received by HUVECs, enhancing the ability of the tube formation of HUVECs and facilitating the angiogenesis around pancreatic cancer. Exosomal lncRNA SNHG11 is involved in the proliferation and angiogenesis of PC 23. Moreover, circ-IARS is up-regulated in PC tissues and plasma exosomes from patients. Exosomal circ-IRAS increases the permeability of the blood vessel lining, thereby promoting vascular metastasis of tumor cells 26. Additionally, MiR-27a plays a role in promoting the growth of multiple cancers. Specifically, exosomes derived from pancreatic cancer cells containing miR-27a have been found to enhance the proliferation, invasion, and angiogenesis in human umbilical vein endothelial cells (HUVECs). This effect is achieved through the negative regulation of a protein called BTG2^49^. Under hypoxic conditions, miR-30b-5p is significantly enriched in PC cell-derived exosomes. These exosomes promote tube formation and the migration of endothelial cells by miR-30b-5p mediating the downregulation of gap junction protein GJA1 50. Exosomal lncRNA UCA1 is transferred into HUVECs, promoting the migration and tube formation of recipient cells. Further research has revealed that lncRNA UCA1 acts as a competing endogenous RNA (ceRNA) for miR-96-5p, leading to the upregulation of AMOTL2 and p-ERK1/2 in HUVECs 18. It is important to note that not all exosomes derived from pancreatic cancer cells promote angiogenesis. For example, MiR-29b is significantly downregulated in BxPC3 and AsPC-1 cells. After transfecting miR-29b mimics into BxPC3 and AsPC-1 cells, exosomal miR-29b can reduce angiogenesis by targeting ROBO1 and SRGAP2^13^. These exosomes are capable of delivering biological molecules into HUVECs and activating their ability to form tube-like structures.

#### Exosomes and Stellate Cells in PC

Cancer-associated fibroblasts (CAFs) are crucial components of the tumor microenvironment. They originate from stellate cells in the PC and play a significant role in the progression of PC. These CAFs create an immunosuppressive microenvironment for PC, making the tumor resistant to external drug treatments 51. Research has shown that exosomes derived from pancreatic stellate cells carry miR-5703 into PC cells. This miRNA downregulates CMTM4 and activates the PI3K/Akt pathway, promoting the proliferation of PC cells 52. In hypoxic conditions, exosomal miR-4465 and miR-616-3p from pancreatic stellate cells (PSCs) target PTEN and activate AKT signaling in PC cells 53. Additionally, exosomes from PSCs carry miR‑21, which induces EMT through the activation of the Ras/ERK signaling pathway 54. Once stellate cells transform into CAFs, exosomes from CAFs carry different substances that regulate the progression of PC. For instance, CAF-derived exosomes deliver miR-421 into PC cells, targeting SIRT3 and regulating the SIRT3/H3K9Ac/HIF-1α axis to enhance the proliferation, migration, and invasion abilities of PC cells 55. Interestingly, exosomes derived from PC cells themselves also promote the conversion of PSCs to CAFs. This creates a positive feedback loop between PC cells and CAFs through exosomal communication, thereby resulting in rapid remodeling of the PC microenvironment and further promoting tumor progression.

#### Exosomes and Bone Marrow Mesenchymal Stem Cell in PC

There is a strong connection between bone marrow mesenchymal stem cells (BM-MSCs) and pancreatic cancer. BM-MSCs play a role in the development of pancreatic cancer through various methods, including the release of exosomes 56. Currently, BM-MSC-derived exosomes have been confirmed to inhibit the malignancy of pancreatic cancer. According to Yao *et al.*, exosomes from BM-MSCs carry circ_0030167 into pancreatic cancer cells and regulate miR-338-5p. This regulation leads to enhanced expression of Wif1, which inhibits the Wnt8/β-catenin pathway. Ultimately, exosomal circ_0030167 suppresses the invasion, migration, proliferation, and stemness of PC cells 57. Another study also supports this conclusion, stating that BM-MSC-derived exosomal circ_6790 facilitates the nuclear translocation of chromobox 7 (CBX7), leading to an increase in the DNA methylation of S100A11 and inhibition of its transcription. As a result, exosomal circ_6790 inhibits the growth, metastasis, and immune escape of PC cells 58. Additionally, miR-1231 has been found to have a potential inhibitory effect on the progression of PC. Exosomal miR-1231 derived from BM-MSCs inhibits the proliferation, migration, invasion, and adhesion to the matrix of PC cells 59. In summary, exosomes derived from BM-MSCs have an inhibitory effect on the development of PC. If these exosomes can be engineered to deliver drugs for PC therapy, they would have advantages over exosomes from other sources. In addition to the inhibitory effect of the drug itself on the progression of PC, the substances present in exosomes would further hinder the progression of PC, thereby improving the effectiveness of engineered exosome-loaded drugs.

#### Exosomes and Lymphocytes in PC

The lymphocyte is one of the main cells to resist the progression of PC. These cells regulate the inflammatory response around PC cells by secreting various active molecules. These immune cells with anti-tumor effects inhibit pancreatic cancer progression by secreting exosomes. Natural killer (NK) cells play a crucial role in the immune system's defense against tumor growth and metastasis. These cells secrete exosomes that are capable of transferring a variety of microRNAs, including miR-16-5p, miR-342-3p, miR-24-3p, miR-92a-3p, and let-7b-5p, into pancreatic cancer cells. Notably, let-7b-5p has been shown to exert an antitumor effect by specifically targeting the gene CDK6, thereby inhibiting cell cycle progression and promoting a tumor-suppressive response. This mechanism highlights the potential of NK cell-derived exosomes as a therapeutic strategy for modulating the tumor microenvironment 60. In another study, miR-3607-3p is found enriched in the exosomes from NK cells and transmitted to PC cells, which will suppress the progression of PC cells via direct targeting of IL-26^61^. However, recent studies suggest that the content of various types of lymphocytes in the pancreatic cancer microenvironment is low, which also leads to the immunosuppressive properties of pancreatic cancer. T lymphocytes are one of the main cells that carry out anti-tumor effects. According to Tao *et al.*, exosomes from pancreatic cancer are taken up by T lymphocytes to activate p38 MAPK, and then induce endoplasmic reticulum stress-mediated apoptosis 62. In general, the secretion of exosomes by lymphocytes can inhibit the progression of pancreatic cancer, but at the same time, exosomes originating from PC cells can mediate the death of lymphocytes. Eventually, immunosuppression was observed in the TME.

### Proliferation of PC cells

The malignant behavior of tumor cells is caused by the abnormal growth of cells. When tumor cells grow rapidly and uncontrollably, they take away essential nutrients from neighboring cells that are necessary for their survival. Cancer-derived exosomes (CDE) and some non-cancer-derived exosomes (nCDE) contribute to the uncontrolled growth of prostate cancer cells. Recent research indicates that exosomes derived from cancer carry various types of genetic material into cancer cells, fueling their proliferation. For instance, CircPDK1 is abundantly expressed in exosomes derived from both pancreatic cancer cells and the serum of patients. The uptake of exosomal CircPDK1 enhances tumor cell proliferation, migration, and glycolysis by sponging miR-628-3p and activating the BPTF/c-myc axis.^25^. Similarly, lncRNA SNHG11 is also highly expressed in the serum of PC patients. Exosomal SNHG11 promotes cell proliferation, migration, and angiogenesis by sponging miR-324-3p and regulating the expression of VEGFA 23. Additionally, exosomal lncRNA 01133 activates the Wnt/β-catenin pathway, leading to the proliferation, migration, invasion, and epithelial-mesenchymal transition (EMT) of PC cells 19.

In addition, exosomes also contain various proteins that promote the proliferation of PC cells. One such protein is ZIP4, a membrane-located zinc ion transporter that regulates, intracellular zinc homeostasis and is upregulated in exosomes from PC cells. Exosomal ZIP4 can significantly promote PC growth 34. Asparaginyl endopeptidase (AEP), highly expressed in solid tumors, is also detected in exosomes from pancreatic cancer. Exosomal AEP contributes to the activation of the phosphoinositide 3-kinase/RAC‑α serine/threonine-protein kinase signaling pathway in PC cells, thereby promoting cell proliferation 63. Lastly, GOT1 is upregulated in PC tissues, and exosomal GOT1 suppresses tumor cell ferroptosis, accelerating PC progression through activating Nrf2/HO-1 axis and upregulating CCR2 expression 35. Notably, these molecules are associated with TNM staging and poor prognosis in PC patients and may serve as early biomarkers for the disease. However, more research is needed to provide additional information and clarify the topic.

### Migration of Pancreatic Cancer Cells

In the early stage of pancreatic tumor metastasis, tumor cells undergo a series of changes in their biological behavior. These changes enhance the metastatic ability and anti-apoptosis of tumor cells 64, 65. Therefore, cancer cells can break away from the primary tumor, invade surrounding tissues, and enter the circulatory system, becoming circulating tumor cells (CTCs) 66. One important process in this progression is called epithelial-mesenchymal transition (EMT). EMT is a reversible process in which epithelial cells lose their polarity and intercellular connections and acquire a more mesenchymal shape 67. During the metastasis and invasion of pancreatic cancer (PC), EMT occurs.^68^. Exosomes, which are small extracellular vesicles, play a role in transmitting information in the tumor microenvironment. Both cancer-derived and non-cancer-derived exosomes can induce changes in the biological functions of tumor cells upon being received. Research has shown that exosomes from pancreatic cancer cells deliver TGF-β1 33 and lncRNA 01133 19 into cancer cells, leading to the induction of EMT. Exosomal lncSox2ot from highly invasive PC cells promotes tumor invasion and metastasis *in vitro* and *in vivo* by enhancing EMT and stem cell-like properties of PC cells 22. MiR-125b-5p is also upregulated in highly invasive PC cells and their exosomes. These exosomes activate MEK2/ERK2 signaling pathway, inducing EMT 69. Linc-ROR, which is highly expressed in PC cells and their exosomes, facilitates EMT in PC cells by activating the HIF1α/ZEB1 signaling pathway and interleukin-1β (IL-1β) 20. Further investigation is needed to determine if these exosomes can serve as biomarkers for the occurrence of EMT in PC.

### Maintenance of Stemness of PC Cells

Tumor stemness maintenance refers to the process of maintaining the characteristics of cancer stem cells. This enables cancer cells to exhibit enhanced migration, proliferation, and resistance to chemotherapy. Exosomes derived from PC cells have been found to promote the stemness of cancers. Wang *et al.* have discovered that hypoxia-induced cancer-derived exosomes transport into tumor cells, thereby promoting the stemness of tumor cells. These exosomal lncROR inhibit the activation of the Hippo/Yes-associated protein (Hippo/YAP) pathway specifically in pancreatic cancer 70. Exosomal lncRNA-Sox2ot has also been reported to promote the stemness of PC cells by acting as a competing ceRNA 22. However, in the progression of PC, some exosomes induce PC stem cell differentiation and suppress stemness maintenance. For instance, miR-21a-5p is upregulated in exosomes derived from M2 macrophages. These exosomal miR-21a-5p molecules specifically target KLF3 and regulate pancreatic stem cells 71. Further research is needed to investigate whether these exosomes, which indicate a strong ability to maintain stemness in PC cells, can serve as biomarkers for predicting the chemoresistance of cancer cells.

## Clinical Application of Exosomes in Pancreatic Cancer

**Figure 3:**
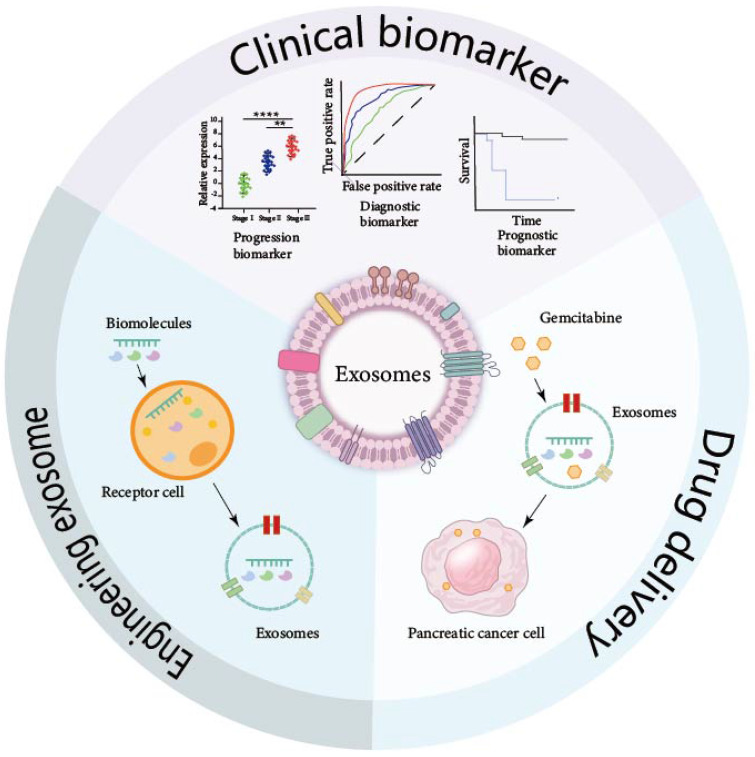
** Clinical application of exosomes in pancreatic cancer.** The unique double-layer membrane of exosomes protects the biomolecules carried in exosomes so that these exosomes carrying special molecules can be maintained in various body fluids for a long time and can be detected and extracted. These exosomes can also be engineered to have a wide range of clinical applications.

Exosomes hold exceptional promise for clinical applications, as depicted in Figure 3. Their unique bilayer membrane structure endows them with the capacity to safeguard their cargo, rendering them resilient to degradation by external factors. The extraction of exosomes from serum, urine, ascites, and other bodily fluids allows for their utilization as diagnostic and prognostic biomarkers. Furthermore, when cells are transfected with small molecules, the exosomes they secrete encapsulate these molecules, offering a vehicle for potential therapeutic agents. By scrutinizing the specific exosomes present in the circulatory system and monitoring the variations in their molecular cargo, the early detection of pancreatic cancer can be achieved. Additionally, the analysis of exosomes provides a valuable tool for assessing the efficacy of treatments, offering a non-invasive and sensitive approach to monitoring disease progression and response to therapy.

### Diagnostic and Prognostic Potential of Exosomes

Early diagnosis plays a crucial role in the treatment of pancreatic cancer. Unfortunately, due to the absence of precise identification methods in the initial stages of the disease, patients often do not receive the most effective treatment when diagnosed. However, there is ongoing research on the potential use of exosomes, which are early indicators, as biomarkers for pancreatic cancer. These exosomes can be found in various bodily fluids and may hold promise for early detection and diagnosis of the disease. Research has investigated that some proteins in exosomes can be detectable in PC.

Exosomes containing specific proteins are often used as biomarkers in pancreatic cancer. For instance, Glypican-1 (GPC1) is a membrane-anchored protein in a variety of cancers including PC. Detecting GPC1-positive circulating exosomes (crExos) is a reliable way to identify early-stage pancreatic cancer, and these GPC1-positive crExos are even more effective as a prognostic marker compared to the current serum biomarker CA 19-9 72. Additionally, combining CD63-GPC1-positive exosomes with CA19-9 shows promising potential for diagnosing resectable pancreatic cancer 73. Another diagnostic biomarker is exosomal ALIX, when combined with CA199, has the potential to detect pancreatic cancer and differentiate between early and advanced stages 74. Exosomal ZIP4 has been found to promote PC growth and serum exosomal. Also, ZIP4 has been identified as a novel diagnostic biomarker for pancreatic cancer 34. Similarly, exosomal-EphA2 has been reported to facilitate cell migration in pancreatic cancer, and levels of serum exo-EphA2 are significantly higher in pancreatic cancer patients compared to those with benign pancreatic disease or healthy controls 75. Additionally, high level of exo-EphA2 expression in pancreatic cancer is linked to a shorter overall survival. This finding indicates that exo-EphA2 can serve as a negative prognostic factor for patients with pancreatic cancer 76, 77. Furthermore, recent research by Alexander Lux *et al.* has shown that the exosomes derived from the serum of pancreatic cancer patients express c-Met and PD-L1. Patients with positive for c-Met and PD-L1 have shown a significantly shorter survival time after surgery 78. Further research has revealed that the levels of PD-L1 in serum-derived exosomes are higher in patients with metastatic pancreatic cancer compared to those with locally advanced disease. Additionally, higher levels of exosomal PD-L1 in patients with advanced pancreatic cancer are associated with worse survival outcomes 79.

The expression of miR-125b-3p, miR-122-5p, and miR-205-5p in plasma exosomes has been investigated as a potential biomarker for PC. A study has confirmed that these miRNAs were overexpressed in the plasma exosomes of PC patients compared to healthy controls 80. Moreover, plasma-derived exosomal miR-19b holds promise as a diagnostic marker for pancreatic cancer 81. Jia Chen *et al.*, have discovered that the expression of miR-451a in serum-derived exosomes from PC patients is significantly upregulated compared to those from patients with pancreatic benign disease and healthy individuals. Furthermore, exosomal miR-451a has been shown to have a significant association with clinical stage and distant metastasis in PC 82. Exosomal miR-1226-3p is another biomarker of PC. The expression of serum exosomal miRNA-1226-3p is downregulated in PDACs compared to benign pancreatic lesions 83. Xiaofan Pu *et al.* have demonstrated that exosomal miR-21 from the plasma of patients with PC is higher compared to the control group. Additionally, exosomal miR-21 can differentiate between patients patients with early-stage PC and healthy controls 84. According to the miRNA microarray analyses, miR-451a has been shown to have the highest upregulation in PC patients at stage II who experienced recurrence after surgery. And exosomal miR-451a shows a significant association with tumor size and stage 85. Another study has indicated that exosomal miR-4525, miR-451a, and miR-21 levels are upregulated in the portal vein blood 86. Research from Lun Wu *et al.* has also reported that exosomal miRNA-21 and miRNA-210 are significantly higher in patients with PC compared to patients with chronic pancreatitis 87. MiR-483-3p is overexpressed in PC and PanIN tissues compared to normal pancreatic duct cells. Circulating miR-483-3p levels are significantly elevated in the serum and serum exosomes of PC patients compared to healthy controls 88. The expression of miR‑23b‑3p is consistently higher in serum exosomes from PC patients as compared to those from healthy controls 89. The expression of exosomal miR-191, miR-21, and miR-451a are significantly up-regulated in patients with PC and IPMN compared to the controls 90. Plasma exosome-derived circRNAs are also investigated to function as biomarkers. Compared to healthy controls, hsa_circ_0006220 and hsa_circ_0001666 are highly expressed in exosomes in the plasma of PC patients, suggesting that these could be utilized as new biomarkers for the diagnosis and treatment of PC 27.

Furthermore, besides using serum-derived exosomes as biomarkers, researchers have also investigated exosomes from other bodily fluids. In patients with advanced pancreatic cancer, it has been observed that exosomes from ascites have a high expression of CD133. However, more research is needed to determine if exosomal CD133 can be used as a biomarker 91. Additionally, in urine exosomes, there is an increased ratio of miR-3940-5p to miR-8069 in pancreatic cancer cases.

According to the research, exosomes from the culture media of PC have shown a significantly higher ratio of miR-3940-5p/miR-8069, and this ratio is higher in urine exosomes than in serum exosomes 92. Another research suggests that messenger RNAs (mRNAs: ARF6 and WASF2) and small nucleolar RNAs (snoRNAs: SNORA25 and SNORA74A) in the serum of patients with PC may be useful for the early detection of PC 93. The presence of miR-196a and miR-1246 in PC exosomes, as determined by qRT-PCR analysis, could potentially serve as indicators of localized PC 94. Exosomal alkaline phosphatase placental-like 2 (ALPPL2), a glycosyl phosphatidyl inositol (GPI)-anchored membrane protein, has also been identified as a potential biomarker for early diagnosis of PC 95. Additionally, cytoskeleton-associated protein 4 (CKAP4), a novel Dickkopf1 (DKK1) receptor, is being investigated as a candidate for PC diagnosis and therapy. CKAP4 is secreted with SEVs from PC cells and the serum CKAP4 levels are higher in patients with PC compared to healthy individuals 96. However, these studies have lacked relevant epidemiological data, and further research is needed.

### Inhibition of exosome release and uptake in Pancreatic Cancer

Pancreatic cancer employs a strategy to promote its progression by releasing exosomes that modulate the functions of various stromal cells, immune cells, and endothelial cells within the tumor microenvironment. This, in turn, facilitates the remodeling of the surrounding microenvironment. Disrupting the release and uptake of exosomes in pancreatic cancer cells presents a promising approach for the treatment of this disease. Yang *et al.* discovered that ZIP4 activates the CREB-regulated expression of RAB27B, which is crucial for the release of exosomes from pancreatic cancer cells. Targeting these pathways may effectively reduce the proliferation of pancreatic cancer cells 97. Additionally, PRKD1 has been identified as a suppressor of motility in pancreatic cancer. Loss of PRKD1 results in decreased phosphorylation of cortactin in pancreatic cancer cells, leading to increased F-actin at the plasma membrane and enhanced exosome release 98.

Wang *et al.* reported that the expression of DUSP2 is significantly suppressed in pancreatic cancer cells, leading to increased secretion of extracellular vesicle-associated VEGF-C 99. In addition, hypoxia has been found to enhance the release and size distribution of exosomes from pancreatic cancer cells which, HIF-1α is a key factor in this process 100. Targeting these molecules could offer new strategies for the treatment of pancreatic cancer.

### Exosomes and the Chemoresistance

Currently, FOLFIRINOX is the primary chemotherapy treatment for pancreatic cancer that has spread locally or metastasized. Modified FOLFIRINOX and combinations of gemcitabine with other drugs are also commonly used 101. Research has shown that exosomes can impact the resistance of pancreatic cancer cells to chemotherapy by regulating their proliferation, EMT, and stemness. According to the research from Mikamori *et al.*, long-term exposure to GEM will increase the miR-155 expression in PC cells and exosomes, which leads to anti-apoptotic activity and GEM resistance in other PC cells 102. Specifically, exosome-derived panc-1 cells that are resistant to gemcitabine (GEM) have been found to increase the GEM resistance of MIA PaCa-2 and BxPC-3 cells. This effect is believed to be mediated by Ephrin type-A receptor 2 (EphA2), a protein that is overexpressed in panc-1 cells 103. Another protein, matrix metalloproteinase 14 (MMP14), a transmembrane Zn2+-dependent MMP, has been found to play a role in promoting recipient cells' resistance to gemcitabine. Exosomal MMP14 is transferred to recipient cells, promoting their sphere formation and migration by enhancing the stability of CD44. As a result, the recipient cells become more resistant to gemcitabine 104. Additionally, exosomes derived from hypoxic pancreatic cancer cells have been shown to promote gemcitabine resistance in normoxic pancreatic cancer cells. This is due to a hypoxia-induced exosomal circular RNA called circZNF91, which competitively binds to miR-23b-3p. This binding leads to enhanced stability of HIF-1α protein and increased glycolysis, both of which contribute to gemcitabine resistance in normoxic cells 29. Hypoxia-induced tumor-derived exosomal lncROR has been shown to enhance stemness and promote chemoresistance in pancreatic cancer cells treated with GEM through the Hippo signaling pathway 70. Interestingly, there is a reciprocal relationship between exosomes and chemoresistance. Prolonged exposure to GEM increases the release of exosomes, which in turn further enhances the chemoresistance in PC 102. Non-oncogenic exosomes derived from cancer-associated fibroblasts (CAFs) also play a role in regulating chemoresistance. These exosomes carry miR-3173-5p into PC cells and promote chemoresistance to GEM by suppressing ferroptosis through the inhibition of ACSL4 105. Moreover, during GEM treatment, certain microRNAs, such as miR-21, miR-181a, miR-221, miR-222, and miR-92a, are highly expressed in exosomes derived from CAFs. These exosomal microRNAs downregulate PTEN expression, leading to increased proliferation and chemoresistance in PC cells 106. CAFs are also involved in the regulation of exosome release by influencing the stiffness of the extracellular matrix 107. As mentioned earlier, exosomes play a critical role in mediating GEM chemoresistance. These molecules are packaged into the exosomes and transferred from chemoresistant cells to sensitive cells, conferring resistance to GEM treatment. According to the research conducted by Ella Rimmer and her colleagues, they have stated that the mentioned effect can be counteracted when GW4869, an inhibitor of exosome biogenesis and release, is present 108. However, it is still necessary to conduct further studies to determine whether the removal of extracellular vesicles during treatment would enhance the response of cells to GEM.

### Engineering Exosomes in Pancreatic Cancer

Engineering exosomes refers to the modification of exosomes by physical and chemical means so that the modified exosomes carry small molecules without changing the structure and characteristics of exosomes. According to the research from Kamerkar *et al.*, exosomes derived from normal fibroblast-like mesenchymal cells were engineered to carry short interfering RNA or short hairpin RNA specific to oncogenic Kras^G12D^. In the mouse models of pancreatic cancer, overall survival is significantly increased after treatment with these exosomes 109. Another research also reports that non-autologous exosomes can deliver CRISPR/Cas9 plasmid DNA which targets the mutant Kras ^G12D^ into recipient pancreatic cancer cells in syngeneic subcutaneous and orthotopic models and suppresses proliferation and tumor growth in these models 42. Chemotherapy drugs can also be transfected into exosomes. Li *et al.* report that GEM is loaded into autologous exosomes which is uptaken by pancreatic cancer cells and significantly suppresses tumor growth in tumor-bearing mice 110.

Exosomes from human umbilical cord mesenchymal stem cells (HUMSCs) are reported to suppress the malignant behavior of tumor cells after being pre-conditioned with exogenous molecules. The expression of Galectin-3 is higher in the pancreatic cancer cells. Xie *et al.* transfected hsa-miRNA-128-3p into HUCMSCs and collected the exosomes from these cells. According to the research, HUCMSC-derived exosomes with hsa-miRNA-128-3p inhibited the proliferation, invasion, and migration of PANC-1 cells *in vitro* by targeting Galectin-3^111^. Another research by Ding et.al, they used HUMSCs exosomes to deliver exogenous miR-145-5p, which suppressed PDAC cell proliferation and invasion and increased apoptosis and cell cycle arrest 112. In the research from Klimova *et al.*, exosomes from human dental pulp MSCs (HD-MSCs) can carry GEM and inhibit the cell growth of pancreatic cancer cells *in vitro*. DP-MSCs were simultaneously engineered to express a suicide gene yCD: UPRT, which will generate 5-fluorouracil (5-FU) in the recipient cancer cells and further suppress the growth of pancreatic cancer cells 113. In brief, engineered exosomes have shown promising application prospects in both *in vivo* and *in vitro* experiments, but there is a lack of relevant clinical trials, and specific research needs to be further carried out.

## Challenges and Perspectives

So far, we have discussed the role of exosomes in pancreatic cancer and their potential clinical application. These exosomes play a pivotal role in regulating the progression of pancreatic cancer cells and remodeling the surrounding microenvironment. Targeting these exosomes offers promising avenues for the treatment of the treatment of pancreatic cancer. However, the study and utilization of pancreatic cancer exosomes face several challenges. Firstly, there is a lack of standard isolation and characterization protocols for pancreatic exosomes 114. The extraction and separation of exosomes are often accompanied by issues such as loss of activity, contamination with non-vesicular substances, and a time-consuming separation process, which are yet to be effectively addressed 115. Moreover, when examining exosomes using electron microscopy, grouping exosomes can lead to overlap among the subsets and other particles, resulting in inaccuracies in experimental results 116. Looking ahead, addressing these challenges will be vital for advancing our understanding and utilization of pancreatic cancer exosomes. Developing standardized protocols, improving isolation and purification techniques, and utilizing more accurate methods for exosome characterization will all contribute to the future success of pancreatic cancer exosome research. In addition, utilizing exosomes as biomarkers for the diagnosis and prognosis of pancreatic cancer poses several challenges. Non-invasive methods, such as extracting exosomes from patient serum, urine, or ascites, offer convenience. However, this approach often requires substantial time and cost investment 117. Moreover, conducting a detailed analysis of specific components within these exosomes becomes more challenging. Loss of exosomes during extraction and contamination with non-vesicular substances further decreases the efficiency of detection. Therefore, developing a cost-effective method for exosome extraction is necessary.

Similarly, engineering exosomes for drug delivery also requires addressing cost and quality issues. The process of loading drugs into extracellular vesicles can lead to greater loss of these vesicles, necessitating accurate assessments of their quality 118. Additionally, further research is needed to determine whether engineered exosomes offer superior advantages over other drug carriers, such as liposomes.

In summary, overcoming these challenges will be crucial for harnessing the full diagnostic and therapeutic potential of exosomes in pancreatic cancer. Developing efficient and cost-effective methods for exosome extraction and engineering, while ensuring their quality, will pave the way for enhanced clinical applications in the future.

## Conclusion

Exosomes, which function as messengers between cells, play a crucial role in regulating the biological processes of cells. This review highlights the significance of exosomes derived from pancreatic cancer cells in the development of pancreatic cancer. Firstly, exosomes derived from pancreatic cancer cells can control the proliferation, EMT, and stenness maintenance of pancreatic cancer cells, and they also impact on surrounding cells. These surrounding cells, in turn, release exosomes that influence pancreatic cancer cells, thus promoting the malignant behavior of pancreatic cancer. Secondly, exosomes derived from pancreatic cancer cells have a regulatory effect on the surrounding tumor microenvironment, creating a suitable environment for the survival and progression of pancreatic cancer cells. Lastly, by examining specific exosome molecules, we can determine if they can be used as potential early markers or prognostic indicators for pancreatic cancer. Pancreatic cancer is an extremely malignant digestive tract tumor, and further exploration of the role of exosomes in this context is warranted.

## Figures and Tables

**Table 1:** Exosomes as Biomarkers in PC.

Category	Source	Content	AUC	Sensitivity	Specificity	Function	Ref.
**Protein**	serum	GPC1	1.000	100%	100%	Diagnostic biomarker for PC	72
**Protein**	serum	ALIX	0.730	53.1%	83.9%	Diagnostic biomarker for PC	74
	serum	ALIX+CA199	0.910	90.6%	83.9%		
**Protein**	serum	ZIP4	0.893			Diagnostic biomarker for PC	34
**Protein**	serum	EphA2				Independent risk factor for PC	76
**Protein**	serum	c-Met	0.779	70%	85%	Negative prognostic factors for PC	78
**Protein**	serum	PD-L1	0.491	14%	94%		
**miRNA**	serum	miR-125b-3p	0.736			Diagnostic biomarker for PC	80
**miRNA**	serum	miR- 122-5p	0.726				
**miRNA**	serum	miR-205-5p	0.829				
**miRNA**	serum	miR-19b	0.942	85.48%	90.57%	Diagnostic biomarker for PC	81
**miRNA**	serum	miR-451a				Diagnostic biomarker for PC	82
**miRNA**	serum	miR-1226-3p	0.740			Negative biomarker for PC	83
**miRNA**	serum	miR-21	0.717			Diagnostic biomarker for PC	84
**miRNA**	serum	miR-10b	0.654			Diagnostic biomarker for PC	
**miRNA**	serum	miR-21+ miR-10b	0.791			Diagnostic biomarker for PC	
**miRNA**	serum	miR-451a				Diagnostic biomarker for PC	85
**miRNA**	serum	miR-4525				Independent prognostic factors for PC	86
**miRNA**	serum	miR-21	0.869	80.00%	90%	Biomarkers and therapeutic targets for PC	87
**miRNA**	serum	miR-210	0.823	83.00%	90%		
**miRNA**	serum	miR-483-3p	0.830	85.7%	72.7%	Diagnostic biomarker for PC	88
**miRNA**	serum	miR‑23b‑3p				Biomarkers and therapeutic targets for PC	89
**miRNA**	serum	miR-191	0.788	71.9%	84.2%	Diagnostic biomarker for PC	90
**miRNA**	serum	miR-21	0.826	80.7%	81.0%		
**miRNA**	serum	miR-451a	0.789	65.6%	86.7%		
**circRNA**	serum	circ_0006220	0.782	77.42%	72.58%	Diagnostic biomarker for PC	27
**circRNA**	serum	circ_0001666	0.806	96.77%	51.61%		
	serum	circ_0006220+ circ_0001666	0.884	74.20%	87.10%		
**Protein**	ascite	CD133				Diagnostic biomarker for PC	91
**miRNA**	urine	miR-3940-5p/miR-8069 ratio	0.732	58.1	89.2	Diagnostic biomarker for PC	92
**mRNA**	serum	WASF2	0.943			Diagnostic biomarker for PC	93
**mRNA**	serum	ARF6	0.940				
**snoRNA**	serum	SNORA74A	0.909				
**snoRNA**	serum	SNORA25	0.903				
**mRNA**	serum	miR-196a				Diagnostic biomarker for PC	94
**mRNA**	serum	miR-1246					
**Protein**	Cell supernatant	ALPPL2				Diagnostic biomarker for PC	95
**Protein**	Cell supernatant	CKAP4				Diagnostic biomarker for PC	96
